# The C3HC type zinc-finger protein (ZFC3) interacting with Lon/MAP1 is important for mitochondrial gene regulation, infection hypha development and longevity of *Magnaporthe oryzae*

**DOI:** 10.1186/s12866-020-1711-4

**Published:** 2020-01-30

**Authors:** Shaoshuai Liu, Yi Wei, Shi-Hong Zhang

**Affiliations:** 1 College of Plant Sciences, Jilin University, Changchun, China; 2 Present address: Institute of Phytopathology, Centre for BioSystems, Land Use and Nutrition, Justus Liebig University, Heinrich Buff-Ring 26-32, D-35392 Giessen, Germany

**Keywords:** C3HC type zinc-finger protein, Mitochondrial Lon/MAP1, Infection hypha development, Mycelia longevity, *Magnaporthe oryzae*

## Abstract

**Background:**

The rice blast is a typical fungal disease caused by *Magnaporthe oryzae*, and the mitochondrial ATP-dependent Lon protease (MAP1) has been proven to be involved in blast development. We previously screened a C3HC type Zinc-finger domain protein (ZFC3), which is interacted with MAP1. The purpose of this research was to study the biological function of ZFC3 protein in *M. oryzae*.

**Results:**

We first confirmed that the ZFC3-RFP fusion protein is localized within the mitochondria. The deleted mutant strains of ZFC3 (*∆ZFC3*) showed the enhanced expression level of mtATP6, particularly mtATP8, and almost unchanged nATP9. Δ*ZFC3* produces more conidia and more tolerance to multiple stressors. The knock-out strain shows more melanin accumulation suggests the susceptibility to aging. Δ*ZFC3* displays faster early-stage hypha infiltration involved in MAP1-mediated pathogenicity in host rice.

**Conclusion:**

These results support the view that ZFC3 is a key regulator involved in gene regulation, stress response, cell wall integrity, longevity, conidiation, infection hypha development and MAP1-mediated pathogenicity in *M. oryzae.*

## Background

*Magnaporthe oryzae* is the best-studied phytopathogenic fungus, which can cause severe blast disease in rice [1, 2]. The disease can lead to a great loss of the yield of grain production during epidemics [3]. The rice blast fungus has a variety of life cycle pathways. It is better to understand the specific function in each pathway, to provide biological evidence in eukaryotic development and pathogenesis with feasible molecular genetic manipulation methods. Since a large number of genes have been well investigated in this pathogen, they supply potential targets for rice blast disease control [4–6].

Transcription factors (TFs) are proteins that bind to specific DNA sequences, which can control the rate of transcription from DNA to mRNA [7, 8]. TFs turn genes on or off to ensure that they are expressed in the right cell at the right time. This TF-controlled regulation occurs throughout the life of the cell and the organism [9–11]. Many putative TFs that specifically associate nuclear matrix and some other related transcriptional factors have been confirmed to globally affect gene activation and repression in the rice blast fungus, and further functional analyses indicated that several TF genes are important for fungal development, pathogenesis, and environmental stress tolerance. For example, in hyphal growth [12, 13], conidiogenesis [14, 15], plant infection [16], response to oxidative stress [17, 18] and longevity [19, 20]. However, fungal cytoplasm transcription factors, particularly mitochondrial gene transcription factors are actually unknown in *M. oryzae*. Therefore, it is necessary to further probe functions of these TFs.

In filamentous fungi, aging research in the early 1950s described that cultures of the filamentous ascomycete *Podosprora anserine* did not grow indefinitely but senesce after a strain specific period of growth [21]. The vital phenotype is characterized by an age-related decrease in the growth rate of mycelium and an increase in the pigmentation of aging cultures [22]. Here are several factors can affect life span: ***i****.* The life span of *P. anserina* was affected after adding different metabolic inhibitors to the medium [23].

Kanamycin and neomycin act as inhibitors of mitochondrial ribosomes could lead to an increased life span [24]; ***ii***. Age-related reorganization of the mitochondrial DNA, nuclear genes and extrachromosomal genetic traits (mitochondrial DNA) can control the onset of senescence [25–27]; ***iii***. Mitochondrial oxidative stress and compensation of mitochondrial dysfunctions [28, 29].

Mitochondria are maternally inherited multifunctional organelles, which can form a comprehensive network in many cells to maintain an intricate balance between fission and fusion, mitochondrial biogenesis, and mitophagy [30–32]. Somatic mitochondrial DNA (mtDNA) mutations and respiratory chain dysfunction accompany normal aging [33], and fungal aging and longevity are highly dependent on mitochondrial integrity and functions [34–37]. Better communication between the nucleus and mitochondria is the basis of different mitochondrial stress signals as well as the nuclear stress response pathways to handle these stressors maintain bioenergetic homeostasis in most cases.

In fungal pathogens, mitochondria play major roles in developmental and morphogenetic switches such as hyphal differentiation and biofilm formation, adaptation to stress, cell wall biosynthesis, and structure, innate immune cell interaction and susceptibility to antifungal drugs [38–42]. Therefore, the mitochondrion is considered a prime target for treating fungal diseases [41, 43].

The ATP-dependent Lon protease is the most highly conserved member of the energy-dependent proteases in a myriad of organisms, which vary across different systems and circumstances [44]. Lon proteases play important biological roles in the cell cycle, differentiation, sporulation, motility, and development during stress [45–48]. MAP1, a Lon-like protease of fungal phytopathogens, shares common functions in response to environmental stressors with fungal pathogens. For example, MAP1 is involved in cell wall integrity and maintains pathogenicity and development of *M. oryzae* [49, 50]*.* The involvement of MAP1 in pathogenesis is through the regulation of specific interacting proteins [49]. Previous research showed that ZFC3 is one of the interacting proteins with MAP1 [50]. The function of nuclear transcription factors have been investigated in mammalian mitochondria and may directly regulate mitochondrial gene expression [51]. There is increasing concern about nuclear transcription factors as a direct regulator of mitochondrial gene expression [52, 53].

In this study, we demonstrate ZFC3 is a TF protein which interacts with MAP1 and localizes to the mitochondria to regulate the relative expression of ATP synthesis genes. *∆ZFC3* knock-out strain is tolerant to stressors and accelerates the aging process of fungi. With the fast infiltration speed in host rice plants, it fails to change the symptoms of the disease. Our findings provide new insights into ZFC3 mediation of the development and metabolism of the fungal pathogen.

## Results

### The structure of the *zfc3* gene in *M. oryzae*

**Fig. 1:**
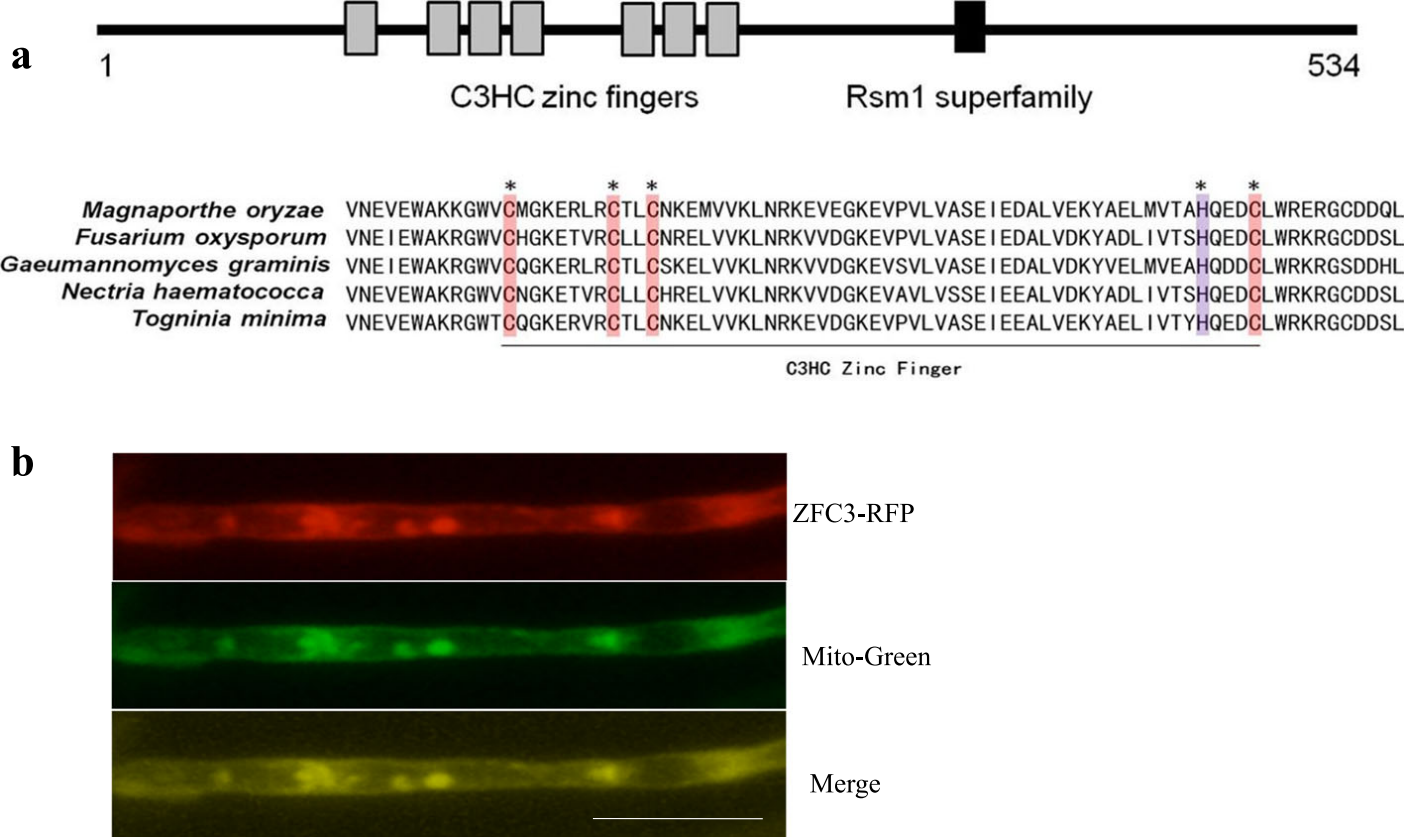
The ZFC3 protein involves two principal domains and localizes to mitochondria. **a** Domain structures of the ZFC3 protein in *M. oryzae* and the conservative and evolutionary analysis of the ZFC3 like protein in representative fungi. **b** Expression and localization of ZFC3-RFP protein in *M. oryzae*. Vegetative hyphae expressing the ZFC3-RFP fusion protein was examined under microscope. Mito-Tracker green (green fluorescent dye as a membrane marker). Scale bar = 2 mm

In different species, the ZFC3 protein contains two conserved domains, namely, the C3HC zinc finger domains and the Rsm1 superfamily domain (Fig. 1a). Based on the conserved amino acid sequence of the *M. oryzae* ZFC3 protein, C3HC zinc fingers conserved domain is common in different species (*M. oryzae*, *Fusarium oxysporum*, *Gaeumannomyces graminis*, *Nectria haematococca* and *Togninia minima*).

The nucleotide sequence of *zfc3* gene (MGG_04317) was aligned and compared with several reported sequences of ZFC3 proteins in other species (Additional file 1: Figure S1a). Phylogenetic analyses of ZFC3 within the eukaryotic tree indicated that this protein was closely related to the other fungal proteins (Additional file 1: Figure S1b).

### Subcellular localization of ZFC3-RFP protein

ZFC3 subcellular localization was determined with a red fluorescent protein (RFP) fusion strategy, then the binary vector was introduced into the wild-type strain. The obtained mutants were used to investigate the cellular localization of ZFC3-RFP fusion protein. The fusion protein was distributed throughout the mitochondria (Fig. 1b) by confocal laser scanning microscopy examination. Indicating that ZFC3 protein mainly localizes to mitochondria in *M. oryzae*.

### Relative expression of ATP synthesis genes increased

**Fig. 2:**
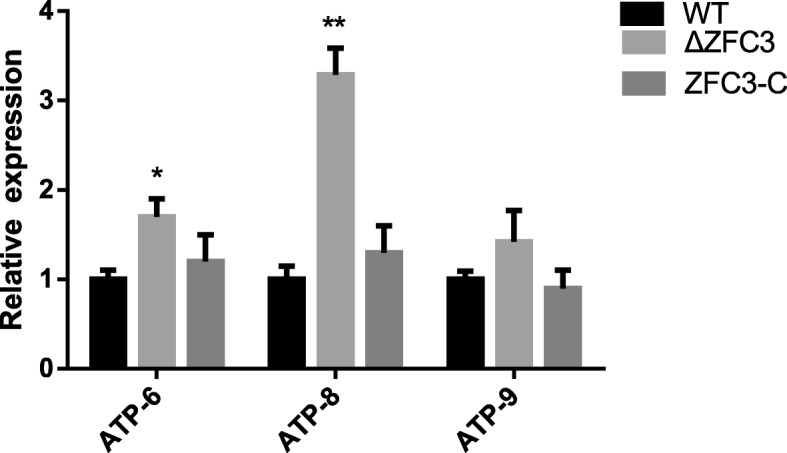
Differential ATP synthesis genes analysis on transcriptomes of the ∆*ZFC3* mutant and those of the WT and complemented strains. The analysis was performed using a one-way ANOVA Tukey’s multiple comparison test. Asterisk (*) indicates a significant difference at *P* < 0.05. All values are normalized to actin. Averages are taken from quadruplicate analysis. Values are based on three biological samples and error bars indicate the mean ± SD

Since the ZFC3 protein localizes to the mitochondria and interacts with the MAP1 protein (Additional file 1: Figure S1c), nuclear and mitochondrial DNA (mtDNA) are thought to be of separate evolutionary origin. Mitochondrial DNA is only a small portion of the DNA and is common in a eukaryotic cell, 15 mtDNA-encoded genes are available in *M. oryzae* (Additional file 2). It has been reported that mtDNA-encoded genes involved in energy metabolism [54] in the process of development. Thus, ATP synthesis gene expression was checked by qRT-PCR. Our data demonstrated that expression level of mtATP6 (GenBank: MGG_21007), particularly mtATP8 (GenBank: MGG_21008) maintained an up-regulated level. The target nATP9 (GenBank: MGG_00892) remained unchanged level (Fig. 2) in comparison with WT and complemented *ZFC3*-C strains. We concluded that ZFC3 involved in ATP synthesis and as a negative regulator of mitochondria.

### Oxidative adaptation and cell wall integrity test

**Fig. 3:**
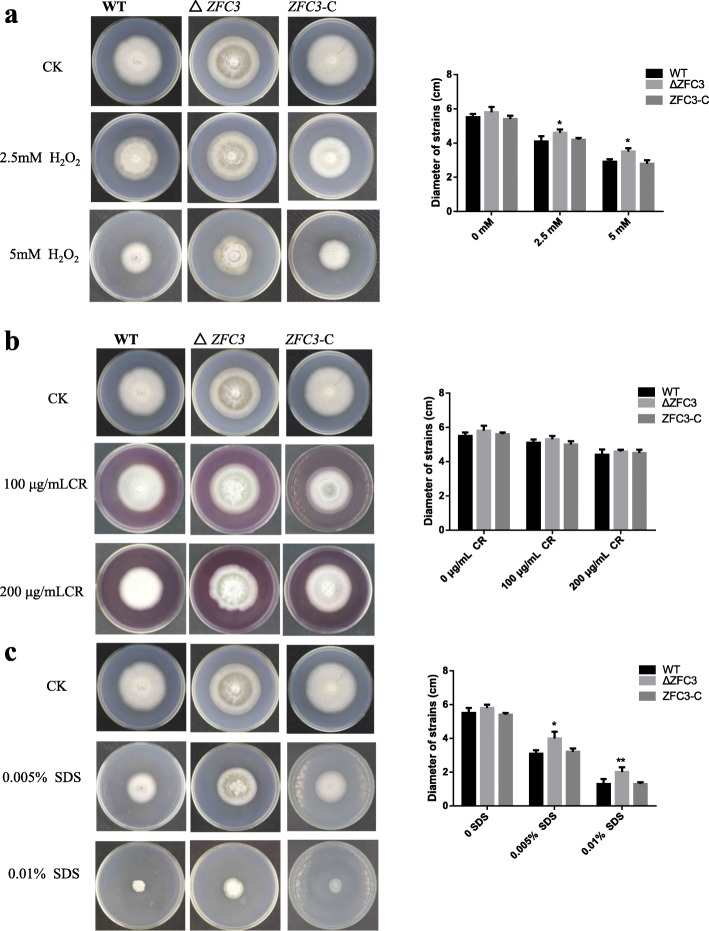
∆*ZFC3* strains are tolerant to oxidative adaptation and cell wall integrity test. **a** WT, Δ*ZFC3* and *ZFC3-*C strains of *M. oryzae* on CM plates supplemented with the oxidative-stress agent H_2_O_2_ (2.5 mM) and H_2_O_2_ (5 mM). **b** The strains inoculated on CM plates containing the cell wall disturbing agents Congo Red (CR, 100 μg/mL) and (CR, 200 μg/mL). **c** The strains inoculated on CM plates containing the cell wall disturbing agents sodium dodecyl sulfate (SDS, 0.005%) and (SDS, 0.01%). Data represent means ± standard deviations (SDs) from three independent experiments in which triplicate plates were examined for each strain in each experiment. **: significant at *P* < 0.01; *: significant at *P* < 0.05

To check the response to oxidative stress in *M. oryzae,* the WT, Δ*ZFC3* and *ZFC3*-C strains were cultured on CM agar containing the oxidative-stress regents H_2_O_2_ at 28 °C for 10 days. Δ*ZFC3* mutant strains displayed increased tolerance to oxidative stress (Fig. 3a). Congo Red (CR) and sodium dodecyl sulfate (SDS) are both typical inhibitors against cell wall synthesis [55–57], and the cell wall disturbing agent’s CR and SDS were used for the cell wall integrity test. The results demonstrated that after 10 days of incubation, there is no significant difference in the growth of CR treatment (Fig. 3b) among different strains. The WT, Δ*ZFC3* and *ZFC3*-C strains also inoculated on CM agar containing the indicated stress-mimetic agent’s SDS, Δ*ZFC3* mutant strains displayed a significant difference to SDS control treatment (Fig. 3c). These data suggest ZFC3 is involved in oxidative-stress adaptation as well as cell wall integrity in *M. oryzae.*

### The absent of ZFC3 accelerates aging

**Fig. 4:**
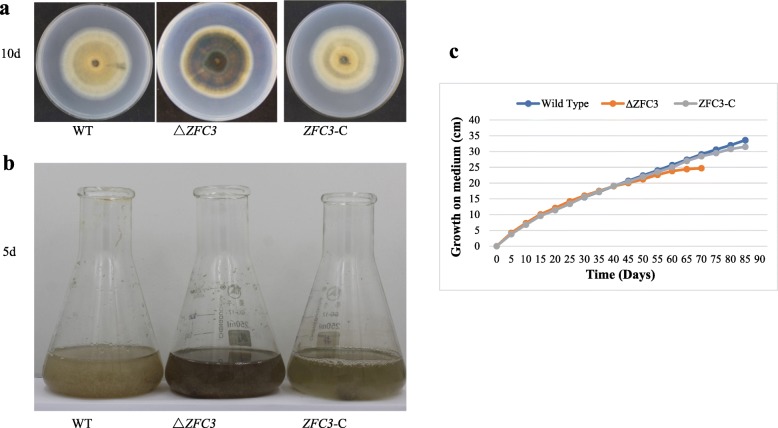
ZFC3 loss accelerates mycelia aging of *M. oryzae*. **a** The loss of *zfc3* gene increased the accumulation of melanin on CM solid medium. **b** The loss of *zfc3* gene increased the accumulation of melanin in CM liquid medium. **c** Life span analysis was carried out among WT, Δ*ZFC3* and *ZFC3*-C strains*.* Data represent means ± standard deviations (SDs) from three independent experiments

Since mitochondrial metabolism increased ATP synthesis, the speed of respiration was enhanced. Normally, the fast respiration rate is appropriately proportional to an increase in metabolic rate. The previous study indicated that differences in metabolic rate have an important effect on the adult lifespan [58]. Traditionally the lowest metabolic rates lived longer and reproduced more often [59]. In order to determine the biological function of *zfc3* gene on lifespan, the wild-type and mutant strains were also inoculated on CM solid plates and CM liquid medium. The extent of melanin production was observed in both different mediums. Δ*ZFC3* mutant showed more accumulation of melanin in both different mediums (Fig. 4a and b). The aging process always accompanied by numerous pigmentary changes, e.g., melanin or lipofuscin may increase with time of age [60]. The result is consistent with the conclusion that the accumulation of melanin means the aging of the organism.

Life span analysis among WT, Δ*ZFC3* and *ZFC3*-C strains was also carried out based on Geydan et al. and Cui et al. [61, 62]. Each strain was separately inoculated on the edge of 150-mm solid plates and cultured in climate chamber for 20 days, then the explants were inoculated on fresh medium again for the next culture cycle. The growth of the Δ*ZFC3* strain quit at the10^th^ day of the fouth culture cycle, indicating the life span of fungi was less than 70 days (Fig. 4c). On the contrary, the WT and *ZFC3*-C strains can grow until the fifth culture cycle. We thus speculated that ZFC3 has a positive effect on fungal lifespan.

### Increased the production of conidia

**Fig. 5:**
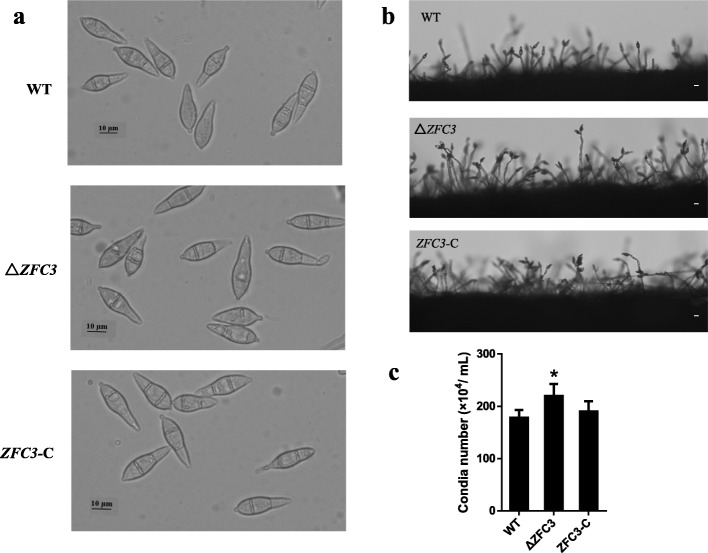
Increased the production of conidia. **a** Light microscopy of conidia produced by the WT, Δ*ZFC3* and *ZFC3*-C strains. Scale bar = 10 μm. **b** Development of conidia is affected by the deletion of *zfc3* gene. Strains grown on SDC medium for 7 days were examined by light microscopy. Bars equal 10 μm. **c** Statistical analysis of conidia production among WT, Δ*ZFC3* and *ZFC3*-C strains. The analysis was performed using an independent samples t-test. Symbol (*) indicates a significant difference at *P* < 0.05. Error bars indicate the mean ± SD from three independent experiments

To investigate the role of ZFC3 in the pathogen conidiation and conidial morphogenesis, we inoculated mycelia on the OM medium and SDC medium. The analysis of conidial morphology revealed that there is no significant difference between Δ*ZFC3* mutant conidia and those of the WT and complemented strains conidia (Fig. 5a). Further analysis of the conidia production, Δ*ZFC3* mutant revealed an increased number of conidia (2.2 ± 0.02 × 10^6^/mL) in comparison to WT (1.8 ± 0.03 × 10^6^/mL) and *ZFC3*-C (1.9 ± 0.05 × 10^6^/mL) (Fig. 5b and c). Taken together, our data demonstrate that ZFC3 controls the production of conidia and as a regulator of the pathogen conidiation.

### The infection hypha of knock-out strains developed earlier than WT and complemented strains, but symptoms remained unchanged

**Fig. 6:**
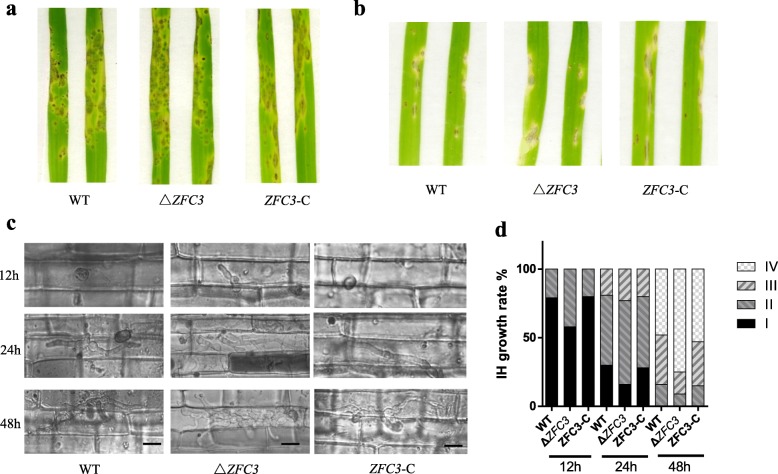
Effect of Δ*ZFC3* mutant on pathogenicity. **a** Rice seedlings cultivar Jijing88 were inoculated by spraying method. Typical leaves were photographed at 7 dpi. **b** Barley seedlings cultivar Golden Promise were inoculated by spraying method. Typical leaves were photographed at 5 dpi. **c** Observation of the invasive hyphal growth inside the rice leaf sheath inoculated with the WT, Δ*ZFC3* and *ZFC3*-C strains, Scale bar = 10 μm (**d**) Statistics of invasive IH hyphal growth rate at 50 appressorium penetration sites by rating the level I to IV. 1 = IH length shorter than 10 mm with no branching; 2 = IH length is 10–20 mm with 0–2 branches; 3 = IH length is longer than 20 mm and/or with more than 2 branches within one cell; 4 = IH has spread to adjacent cells

To comprehensively evaluate the virulence role of Δ*ZFC3* in *M. oryzae*. we inoculated intact host rice leaves (compatible cultivar JJ88) and barley cultivar Golden Promise with conidial suspension (4 mL, 5 × 10^4^ spores/mL) by the spraying inoculation method [63]. Lesions caused by the WT and mutant strains were observed at 7 d (rice) and 5 d (barley). The control and Δ*ZFC3* mutant strains produced obvious lesions on rice as well as barley leaves and displayed the same infection symptom of all strains (Fig. 6a and b).

To elucidate the difference in the process of infection, we performed an infection assay to examine the early infectious hyphae growth on rice leaf sheaths. Infectious hyphae growth was assayed on rice tissues at 12, 24 and 48 hpi post-inoculation using spore suspensions. Δ*ZFC3* displayed faster infection speed compared to WT and complemented strains (Fig. 6c and d). These results suggested that *zfc3* was essential for early penetration and infectious growth in *M. oryzae*. The production of melanin significantly enhances the virulence of many important human and plant pathogenic fungi, so fungal melanin also plays an important role in disease spreading [64–66], thus explaining why Δ*ZFC3* mutant with more melanin accumulation displayed faster hypha infiltration in host rice.

## Discussion

The rice blast fungus *M. oryzae* is a typical model between fungal-plant interaction. A large number of important genes have been characterized for functional analysis. They are mostly involved in the process of fungi’s development and physiology. TF protein ZFC3 is an interacting protein of Lon protease. MAP1 is a member of Lon protease involved fungal pathogenesis. In this study, we set out to explore the role of ZFC3 mediated by MAP1 in the development and pathogenicity of the fungus *M. oryzae*, to provide the evidence of TF roles in Mitochondria.

A large number of ZFC3 orthologs have been characterized in the fungal pathogen. Moreover, many studies have confirmed that ZFC3 can act as mitochondrion protein (Fig. 1b) and play an important role in the fungi’s development and metabolism. Bioinformatics analysis revealed that ZFC3 protein is present in several filamentous fungi, including GgZFC3 (*Gaeumannomyces graminis*, XP_009223819.1), CoZFC3 (*Colletotrichum orbiculare*, TDZ25575.1), FgZFC3 (*Fusarium oxysporum,* EXL95675.1), VmZFC3 (*Valsa mali* KUI69614.1), BbZFC3 (*Beauveria bassiana* XP_008599184.1), VdZFC3 (*Verticillium dahlia* XP_009650679.1). These filamentous fungi share the conserved C3HC zinc finger domains which are a vital part of the genome. To clarify the biological function of ZFC3, we generated gene deletion mutant with the gene homologous recombination method and complemented mutant, as well as the red fluorescent protein-tagged mutant to determine the subcellular location of ZFC3 in *M. oryzae*. Confocal results showed that ZFC3 localized within the mitochondria. The interacting protein MAP1 was also localized in the mitochondria [49]. A previous study showed that MAP1 was also important in maintaining the healthy lifespan of the filamentous fungus *Thermomyces lanuginosus* [62]. Thus, we speculate ZFC3 works in conjunction with MAP1 to maintain mitochondrial integrity and functions in fungi. In the Δ*ZFC3* mutant, the relative expression of ATP-synthesis related genes is up-regulated (Fig. 2). ZFC3 involves the metabolic of organism mediated by bioenergetics and biosynthetic pathways to maintain the function of mitochondria to keep the organism in healthy condition for growth and development.

Mitochondria is a major organelle to generate oxidative stress. Due to its role in converting oxygen and nutrients into adenosine triphosphate (ATP), it is generally reported to be involved in oxidative-stress adaption [33, 49]. The tolerance of *M. oryzae* increased when the WT and knock-out strains were exposed to different concentrations of H_2_O_2_ (Fig. 3a). Additionally, the Δ*ZFC3* strain was also tolerant to SDS treatment (Fig. 3c) in comparison with WT and complemented strains. These results indicate that ZFC3 has a specific role in dealing with oxidation stress as well as cell wall integrity.

Melanin ranks as one of the important natural pigments, as it is synthesized by members of all biological kingdoms. It is normal in a variety of species, such as fungi, bacteria, and helminths. Melanin is a sign of aging with the accumulation of pigment. Besides, it has broad contributions to fungal pathogenesis [67–69]. Therefore, the increased production of melanin in Δ*ZFC3* mutant (Fig. 4a and b) indicates the accelerated aging and the enhanced early stage of pathogenicity in *M. oryzae*.

For pathogen inoculation experiments, we observed that the Δ*ZFC3* mutant exhibited the same symptom of the infection leaves. It did not change the virulence of *M. oryzae,* but the increased amount of the conidia production (Fig. 6a and b) and the fast hypha infection rate (Fig. 6c and d) are different from the WT and complemented strains. The increased ATP synthesis suggests a high energy consumption for hypha infiltration. Higher metabolic rates increased free radical formation, which in turn may accelerate aging and lead to early mortality [70, 71]. Δ*ZFC3* mutant exhibited enhanced development phenotypes, such as increased hyphal growth rates and conidium production (Fig. 5b and c). Indicating that *zfc3* is involved in the regulation of conidiation and infection-related development. It also highlights the importance of the transcription factors for pathogenicity [12, 18].

We further will clarify the function and regulation mechanisms of the zinc-finger protein, to confirm the transcription activity of ZFC3 protein and to identify the promoter or non-specific mtDNA sequences, and mtRNA sequences by ChIP and RIP (RNA-IP) approaches. These may constitute a research direction to reveal the mysterious veil of this transcription factor.

## Conclusions

In short, this study provides many new insights into ZFC3 on the regulation of asexual development, growth, conidiation, pathogenicity, and especially aging in *M. oryzae*. Moreover, this study establishes a solid basis in *M. oryzae* to further explore the specific mitochondrial metabolism process as regulated by ZFC3.

## Methods

### Strains and culture conditions

The *M. oryzae* JL0910 strain used as the wild-type (WT) was isolated and purified from *Oryza sativa* cultivar Jijing88 (a widely planted variety in Jilin Province, China), the spring barley (*Hordeum vulgare* L.) cv. Golden Promise was used in the experiment, the rice and barley plants were grown in a climate chamber under 16 h light photoperiod (240 μmol m^− 2^ s^− 1^ photon flux density) at 18 °C/14 °C (day/night). All fungal strains were kept on paper filters at − 20 °C in our lab. An oatmeal agar medium (OM, 4% (w/v) oatmeal, 2.0% (w/v) agar) and corn agar media (SDC: 100 g of straw, 40 g of corn powder, 15 g of agar in 1 L of distilled water) at 25 °C under bright light [63, 72] was used for sporulation analysis and conidia harvesting, genomic DNA isolation, transformation, measurements of vegetative growth rate and conidiation as described [73]. For testing sensitivities to various stresses, fungal growth was determined after culturing at 22 °C on complete medium (CM:10 g/L glucose, 2 g/L peptone, 1 g/L yeast extract, 1 g/L casamino acids, 0.1% (V/V) trace elements, 0.1% (V/V) vitamin supplement, 0.5 g/L MgSO_4_, 6 g/L NaNO_3_, 0.5 g/L KCl, 1.5 g/L KH_2_PO_4_, pH 6.5) plates. Each test was repeated three times.

### Nucleic acid manipulation

For generating the *zfc3* gene replacement construct pXEH20, the upstream and downstream fragments of the *zfc3* gene were respectively amplified with primers CL-S/CL-A and CR-S / CR-A (Additional file 3). The resulting PCR products were cloned into the *XhoI* - *EcoRI* and *BamHI* - *HindIII* sites of vector pXEH20. The knockout vector was introduced into *Agrobacterium tumefaciens* strain AGL-1 and then do transformation with *M. oryzae* JL0910 by the ATMT method as described [74], Transformants cultured in hygromycin at 200 μg/mL were screened, then they were identified by PCR with primers HYG-F/HYG-R (Additional file 3). To generate complemented vector, a fragment containing 1800 bp upstream of the coding region of *zfc3* was amplified by PCR with primers pZFC3-S/pZFC3-A (Additional file 3) and cloned into vector pCB1532 [49]. The complementary vector was transformed into *A. tumefaciens* strain AGL-1 and the resultant transformants generated by the ATMT method were screened on chlorimuron-ethyl containing DCM medium. Selected transformants were determined by diagnostic PCR to confirm the integration cases, so *ZFC3*-C mutant strains were also taken as control.

### RT-PCR and qRT-PCR analysis

Total RNAs were isolated from mycelia harvested from 2-day-old CM media with TRIzol reagent (Invitrogen) and purified with the DNA-free kit (Ambion). The first-strand cDNA was synthesized from one microgram of total RNA using the Improm II RT-PCR kit (Promega, Madison, WI). The designed PCR and RT-PCR primers (Additional file 3) were used for amplifying the full length of *zfc3* DNA and cDNA from reverse transcription. The ABI Prism 7500 Sequence Detection System (Applied Biosystems) and SYBR® *premix EX taq*™ II (TliRNaseH Plus) Kit (Takara, Dalian, China) were used for qRT-PCR analysis with primers (Additional file 3). The *M. oryzae* actin gene (MGG_03982.6) was taken as a reference gene for normalization.

### Generation of gene fluorescent fusion sub-cellular localization mutants

The pKD7 plasmid vector (a kind gift from Dr. Wang Hongkai and Dr. Jianping Lu, Zhejiang University, China) including *DsRed* gene was applied for transformation. The localized fragments, which contained the entire targeted gene was amplified by PCR with primers (DSRED-F/DSRED-R) (Additional file 3) and integrated into pKD7 vector. The localized vector was introduced into *A. tumefaciens* strain AGL-1 and then do transformation with *M. oryzae* JL0910 by the ATMT method [74]. Transformants cultured in G418 at 200 μg/mL were screened. To detect mutants by PCR with primers NEO-pl/NEO-p2 (Additional file 3).

### Lifespan measurement

Lifespan test was based on the method of [61, 62] with suitable optimization. In a word, lifespan was determined in time (days) and in length (cm) of continuous growth on Petri dishes (150 mm × 25 mm) filled with 65 mL CM medium. Each experiment was carried out at least two replications. The stains were incubated under an angle of 30°–45° and to record the growth every 2 days. Explants were transferred to fresh medium for further measurement form the edge of over-grown Petri dishes. The observation was done until the growth rate declined significantly. They were classified as non-senescent when the growth rate did not slow down and when there were no morphological changes. Fast senescing cultures displayed a decline in the growth rate and morphological changes and accompanied by a cessation of growth.

### Sporulation formation, rice sheath penetration assays and plant infection assays

Quantitative measurement of condition was assayed on OM medium and SDC medium, while the aerial hyphal and conidial development was monitored as described previously [75].

Plant infection assays were performed on 4-week-old rice seedlings (*Oryza sativa* cv. JJ88) and barley cultivar Golden Promise by spraying 4 mL of the conidial suspensions (5 × 10^4^ conidia/mL in 0.2% gelatin). Inoculated plants were placed in a moist chamber at 28 °C for the first 24 h in darkness, and then placed in another moist chamber with a photoperiod of 12 h under the light. The disease severity was assessed after inoculation.

Conidial suspensions were injected into seedling leaf sheaths by a 1-mL syringe, 100 μL of conidial suspension (5 × 10^4^ spores/mL) on the inner leaf sheath cuticle cells. and the inoculated plants were placed in a moist chamber. Lesion formation and necrosis around the inoculation sites were examined when the injection-wounded leaves unfolded at different time points after injection. Mean IH growth rates and movement to adjacent cells at 12, 24 and 48 hpi were determined from 50 appressoria per treatment, repeated in triplicate, as previously described [75].

### Microscopy examination

Live-cell imaging was performed as described previously using 3 cm-long leaf sheaths segments from around 3-week-old rice plants and injecting one end of the sheath with a spore suspension of 1 × 10^5^ spores/mL in 0.2% gelatin. At the indicated time points, leaf sheaths were trimmed and observed using a Nikon Eclipse 80i microscope.

In order to examine the subcellular localization of the ZFC3 protein, subcellular localization mutants were cultured with CM medium and the hypha was harvested 5 days later. The hyphae were washed 3 times with ddH_2_O and placed in100 nM Mito-green solution for 30 min. Mito-Green (Invitrogen, Ltd., Paisley, United Kingdom) is a carbocyanine-based and mitochondrion-selective green fluorescence regent, which can be used for staining and tracking the presence of mitochondrion location under the 488 nm laser wavelength with Olympus Xa21 microscope (Olympus, Tokyo, Japan).

### Statistical analysis

All experiments were performed three times. The means ± SD of the growth rate and relative expression were decided using SPSS statistics 22 software, *P* < 0.05 was considered statistically significant. Error bars mean standard deviation.

## Supplementary information

**Additional file 1: Figure S1.** Phylogenetic and structural analysis of ZFC3 and its homologs. (a) Sequence alignment of *zfc3* in different species of fungi. (b) The phylogenic tree was drawn using MEGA7. GgZFC3 (*Gaeumannomyces graminis*, XP_009223819.1), CoZFC3 (*Colletotrichum orbiculare*, TDZ25575.1), FgZFC3 (*Fusarium oxysporum,* EXL95675.1), VmZFC3 *(Valsa mali* KUI69614.1), BbZFC3(*Beauveria bassiana* XP_008599184.1), VdZFC3*(Verticillium dahlia,* XP_009650679.1). Phylogenetic tree based on the C3HC zinc fingers domain from different eukaryotic organisms indicating that ZFC3 has a relatively close relationship with the fungi group. (c) Protein-Protein interaction between MAP1 and ZFC3. Yeast two-hybrid analysis of MAP1and ZFC3 interaction. Test: interaction between MAP1 and ZFC3 candidate; CK0: self-activation controls; CK^+^: positive controls; CK^−^: negative controls.

**Additional file 2 Figure S2.** The mtDNA-encoded genes in mitochondria organelle and a nuclear-encoded ATP synthesis gene in *M.oryzae.*

**Additional file 3: Table S1**. Primers used for experiments.

## Data Availability

All data generated and/or analyzed during the current study are included in this published article (and in its supplementary files). The datasets used and/or analyzed during the current study are available from the corresponding author on reasonable request.

## Abbreviations

Lon/MAP1: ATP-dependent Lon protease
OM medium: Oatmeal medium
qRT-PCR: Quantitative reverse transcription polymerase chain reaction
SDC medium: Straw decoction and corn powder medium
ZFC3: C3HC type Zinc-Finger Protein
