# Supplementary figures and images for: The C3HC type zinc-finger protein (ZFC3) interacting with Lon/MAP1 is important for mitochondrial gene regulation, infection hypha development and longevity of Magnaporthe oryzae
Source: BMC Microbiol. 2020 Jan 30;20:23. doi: 10.1186/s12866-020-1711-4 (PMC6993355; doi:10.1186/s12866-020-1711-4)

## Slide 1
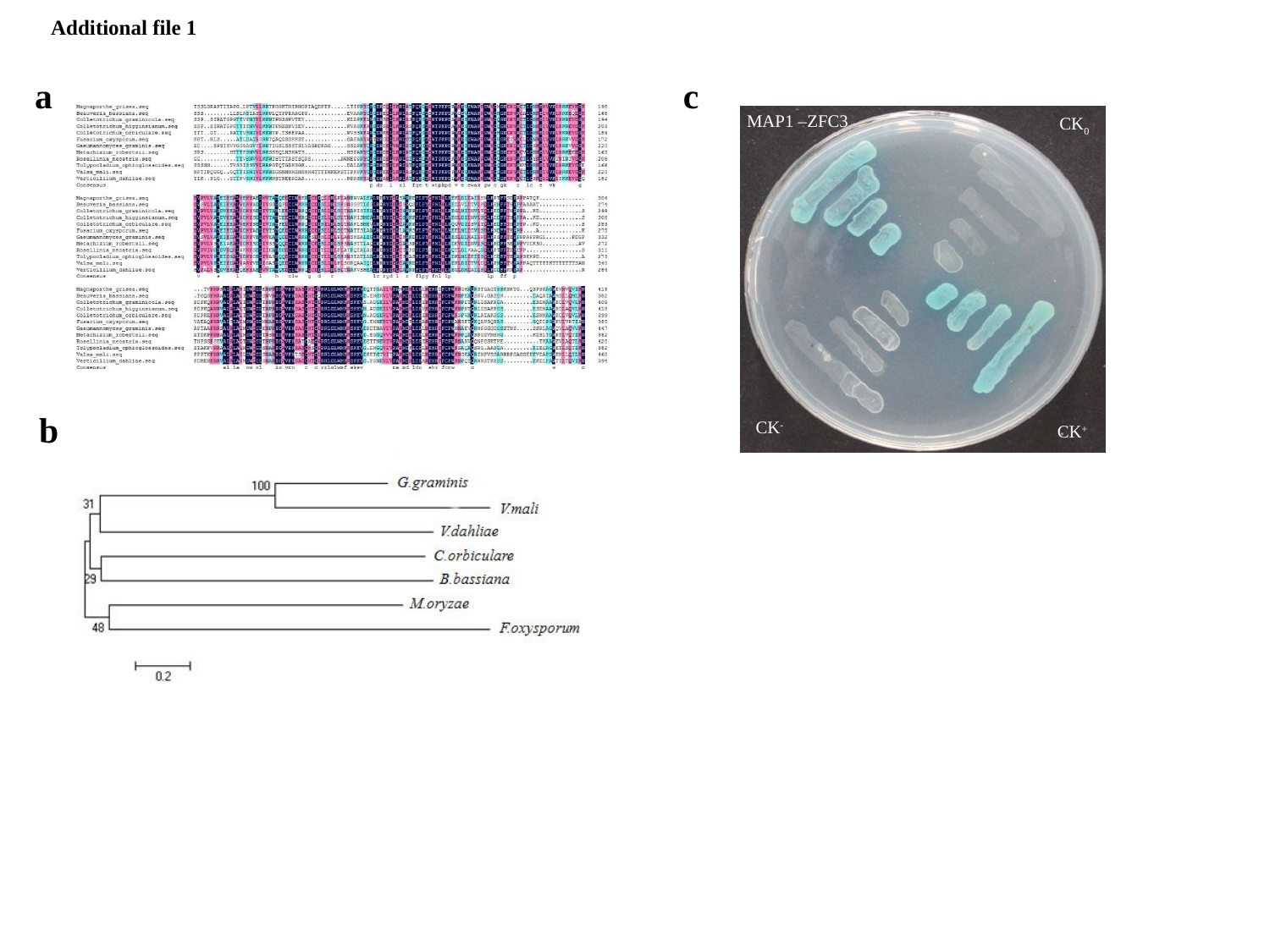

Additional file 1
a
b
c
MAP1 –ZFC3
CK0
CK-
CK+

Supplement: Supplementary file 1 — Additional file 1: Figure S1. Phylogenetic and structural analysis of ZFC3 and its homologs. (a) Sequence alignment of zfc3 in different species of fungi. (b) The phylogenic tree was drawn using MEGA7. GgZFC3 (Gaeumannomyces graminis, XP_009223819.1), CoZFC3 (Colletotrichum orbiculare, TDZ25575.1), FgZFC3 (Fusarium oxysporum, EXL95675.1), VmZFC3 (Valsa mali KUI69614.1), BbZFC3(Beauveria bassiana XP_008599184.1), VdZFC3(Verticillium dahlia, XP_009650679.1). Phylogenetic tree based on the C3HC zinc fingers domain from different eukaryotic organisms indicating that ZFC3 has a relatively close relationship with the fungi group. (c) Protein-Protein interaction between MAP1 and ZFC3. Yeast two-hybrid analysis of MAP1and ZFC3 interaction. Test: interaction between MAP1 and ZFC3 candidate; CK0: self-activation controls; CK+: positive controls; CK−: negative controls. [file 12866_2020_1711_MOESM1_ESM.pptx]
