# Supplementary material for: The C3HC type zinc-finger protein (ZFC3) interacting with Lon/MAP1 is important for mitochondrial gene regulation, infection hypha development and longevity of Magnaporthe oryzae
Source: BMC Microbiol. 2020 Jan 30;20:23. doi: 10.1186/s12866-020-1711-4 (PMC6993355; doi:10.1186/s12866-020-1711-4)
Supplement: Supplementary file 2 — Additional file 2 Figure S2. The mtDNA-encoded genes in mitochondria organelle and a nuclear-encoded ATP synthesis gene in M.oryzae. [file 12866_2020_1711_MOESM2_ESM.pptx]

## Slide 1
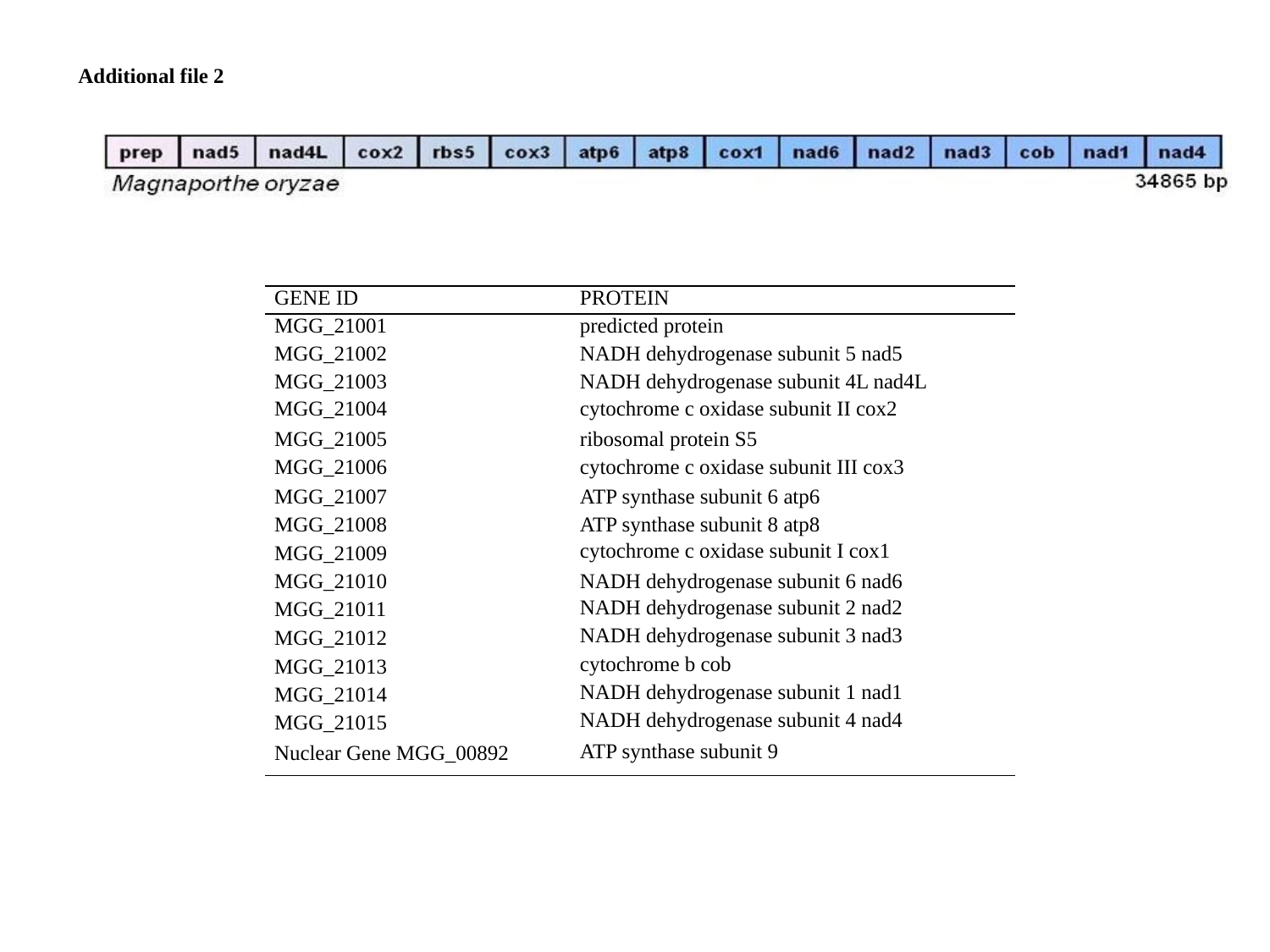

Additional file 2
| GENE ID | PROTEIN |
| --- | --- |
| MGG\_21001 | predicted protein |
| MGG\_21002 | NADH dehydrogenase subunit 5 nad5 |
| MGG\_21003 | NADH dehydrogenase subunit 4L nad4L |
| MGG\_21004 | cytochrome c oxidase subunit II cox2 |
| MGG\_21005 | ribosomal protein S5 |
| MGG\_21006 | cytochrome c oxidase subunit III cox3 |
| MGG\_21007 | ATP synthase subunit 6 atp6 |
| MGG\_21008 | ATP synthase subunit 8 atp8 |
| MGG\_21009 | cytochrome c oxidase subunit I cox1 |
| MGG\_21010 | NADH dehydrogenase subunit 6 nad6 |
| MGG\_21011 | NADH dehydrogenase subunit 2 nad2 |
| MGG\_21012 | NADH dehydrogenase subunit 3 nad3 |
| MGG\_21013 | cytochrome b cob |
| MGG\_21014 | NADH dehydrogenase subunit 1 nad1 |
| MGG\_21015 | NADH dehydrogenase subunit 4 nad4 |
| Nuclear Gene MGG\_00892 | ATP synthase subunit 9 |
