# Supplementary material for: The C3HC type zinc-finger protein (ZFC3) interacting with Lon/MAP1 is important for mitochondrial gene regulation, infection hypha development and longevity of Magnaporthe oryzae
Source: BMC Microbiol. 2020 Jan 30;20:23. doi: 10.1186/s12866-020-1711-4 (PMC6993355; doi:10.1186/s12866-020-1711-4)
Supplement: Supplementary file 3 — Additional file 3: Table S1. Primers used for experiments. [file 12866_2020_1711_MOESM3_ESM.pptx]

## Slide 1
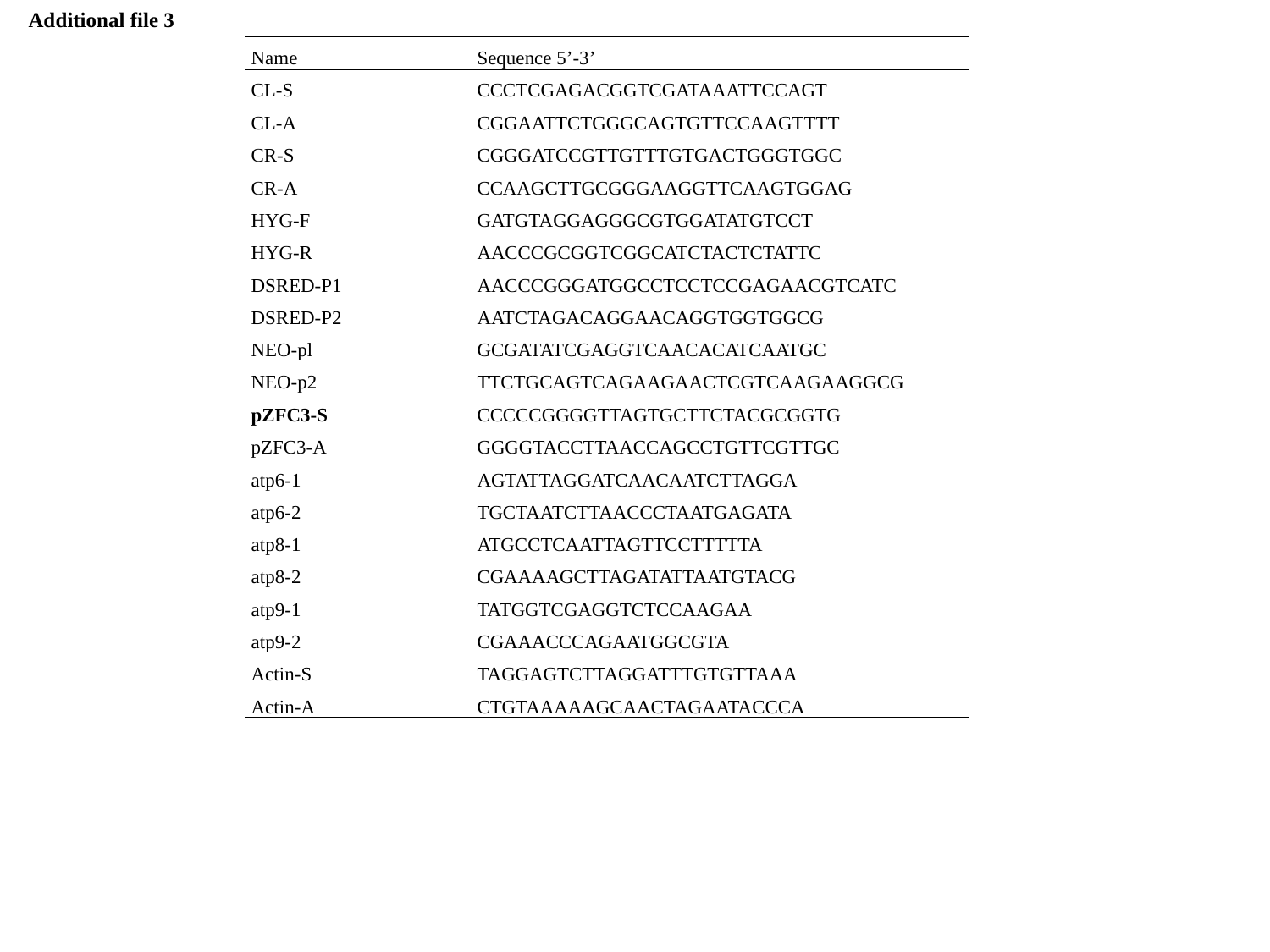

Additional file 3
| Name | Sequence 5’-3’ |
| --- | --- |
| CL-S | CCCTCGAGACGGTCGATAAATTCCAGT |
| CL-A | CGGAATTCTGGGCAGTGTTCCAAGTTTT |
| CR-S | CGGGATCCGTTGTTTGTGACTGGGTGGC |
| CR-A | CCAAGCTTGCGGGAAGGTTCAAGTGGAG |
| HYG-F | GATGTAGGAGGGCGTGGATATGTCCT |
| HYG-R | AACCCGCGGTCGGCATCTACTCTATTC |
| DSRED-P1 | AACCCGGGATGGCCTCCTCCGAGAACGTCATC |
| DSRED-P2 | AATCTAGACAGGAACAGGTGGTGGCG |
| NEO-pl | GCGATATCGAGGTCAACACATCAATGC |
| NEO-p2 | TTCTGCAGTCAGAAGAACTCGTCAAGAAGGCG |
| pZFC3-S | CCCCCGGGGTTAGTGCTTCTACGCGGTG |
| pZFC3-A | GGGGTACCTTAACCAGCCTGTTCGTTGC |
| atp6-1 | AGTATTAGGATCAACAATCTTAGGA |
| atp6-2 | TGCTAATCTTAACCCTAATGAGATA |
| atp8-1 | ATGCCTCAATTAGTTCCTTTTTA |
| atp8-2 | CGAAAAGCTTAGATATTAATGTACG |
| atp9-1 | TATGGTCGAGGTCTCCAAGAA |
| atp9-2 | CGAAACCCAGAATGGCGTA |
| Actin-S | TAGGAGTCTTAGGATTTGTGTTAAA |
| Actin-A | CTGTAAAAAGCAACTAGAATACCCA |
